# Effects of Fear

**Published:** 1848-07

**Authors:** 


*Effects of Fear.*—The Journal de la Société de Medicine de Montpellier details two very curious instances in which the effects of fear had been strange and widely different, in one case tending to cure, and in the other fatal. A man thirty-five years old, was just going to be operated upon for strangulated inguinal hernia, when all the usual means for reduction had failed. Just as M. Cabarret was making the cutaneous incision he perceived the patient turning pale ; respiration became very slow, and he was soon covered with a profuse cold perspiration ; eyes staring, and the whole countenance haggard. But how great was the surgeon's astonishment when he perceived the sudden diminution of the tumour. Reduction had taken place during the shock produced by fear. A few drops of ether revived the patient, and in two days afterwards he was perfectly well. The second case refers to a young man twenty-three years of age, of a lymphatic temperament, and whose cervical glands were tumefied. Having received a challenge to fight a duel, which he had accepted, immediately on returning home he complained of headach, and went to bed, where he remained in a state of great prostration for four days, without answering any questions. On the fourth day, inflammatory fever came on, and on the eighth, he died in a low typhoid state. Intelligence remained clear to the last, and no complaint of any pain whatever was made.—*Ibid.*

